# Injuries to the Fingers

**Published:** 1892-04-16

**Authors:** 


### INJURIES TO THE FINGERS.

No part of the body is so frequently injured as the fingers, and no part (except, perhaps, the eye) is of such importance to those who have to gain their livelihood by manual work. We ought, therefore, to look upon a crushed or lacerated digit with as much care as upon a serious accident to the leg. The mere fact, however, that these accidents are so common often makes the surgeon somewhat careless, and this can easily be understood by anyone who has seen the crowds of men with bandaged hands that attend the out-patient department of any large hospital. Yet nothing so well repays scrupulous care in treatment as these cases. There are two or three points in connection with the subject which are worth special consideration. Firstly, as to the primary dressings to be applied in bad wounds of the fingers. Simple, clean cut wounds need not be considered; all that is necessary is to keep the edges together and to ensure cleanliness. It is, of course, of the utmost importance to remove foreign bodies, such as bits of glass, dust, pieces of wood, &c. This may be done by well soaking the part in a hot solution of carbolic, not made by putting a "1 to 20" mixture with hot water, but by adding the hydrated phenol (containing 10 per cent. of water) to hot water. This serves a twofold purpose; it acts as a local anæsthetic, and enables foreign particles to be removed more easily, and it is the best means of rendering the wound aseptic. It should, however, be of the strength of 1 in 20, not weaker.

A few deep and strong sutures should be inserted if necessary, silk being perhaps better than horsehair or silk-worm gut, as the two last are apt to cut the parts when swelling occurs. A horsehair drain should always be introduced. If bleeding has ceased the wound should be well sprinkled with aristol or crystalline iodoform. It is usual to put boracic lint dipped in water next to the skin, and this is a good dressing; but it must be remembered that in a short time this will probably stick very firmly and unless you can be quite certain of primary union (which can rarely occur), this causes great pain from retaining serous discharges, &c., and when the wound is dressed the second time, the removal of the dried and hard lint causes the greatest torture, often causing strong men to feel faint. This may be obviated by putting a little "green protective" next to the wound, and then swathing the finger in antiseptic wool or lint. A splint is generally advisable in severe lacerations, but it should be a long one, or the whole hand should be included. An ordinary finger-splint often does more harm than good. The hand and arm should be put up in a triangular sling, the elbow being well supported as well as the whole length of the forearm.

If treated in this way the pain is not usually very severe, but sometimes it becomes intense, in spite of every care, especially if cellulitis follows. In such cases a dose of chloral hydrate (gr. 15 or so) should be given at bed-time and repeated, if necessary. The occasional severity of the pain can easily be accounted for; the nerves lie in a dense unyielding tissue for the most part, and owing to the large number of Pacinian and touch corpuscles given off from them, the perineurial sheath is thick; for these reasons the least tension may give rise to great pain. The tendon is often a source of trouble, for if seriously injured, and not removed when first dressed, it is apt to slough away slowly, acting as a foreign body and keeping the wound open. If there is any chance of saving it, the injured end (supposing it to be severed) may be stitched on to the theca or even the skin; but otherwise it should be carefully trimmed, and any ragged portions removed.

The nail is also a source of difficulty. If only partially destroyed, the dead part causes great irritation, and the end of the finger seldom heals up until the offending piece has been removed. Injury to the periosteum readily recovers if the wound can be kept aseptic, and even compound fractures of the phalanges are recovered from, and leave sometimes useful fingers. It is difficult in fact to limit the extent to which conservative surgery of the fingers can be carried, and the patient who obstinately refuses an amputation, frequently triumphs by saving a fairly useful member. The power of recovery in all parts of the hand is marvellous.

It is a common accident for the ends of the fingers to be chopped off in straw and hay-cutting machines, &c. These cases ultimately do well, but the healing is lengthy, owing to the want of skin flaps. Small skin grafts do not seem to readily grow on the granulating surface, and the epithelium does not spread from the sound edge, partly, perhaps, because of the sharp angle it has to turn in order to get on to the raw surface. Strapping cross-ways over the end of the finger is useful in such cases; on removal of the plaster, the blue, freshly-grown epithelium can be seen spreading over the protected part.

*Amputations of the fingers* for injury do not always heal kindly, although it has been stated that they do so "with exceptional rapidity." Unfortunately, asepsis is sometimes impossible, and the result is cellulitis, "giving" of the stitches, and an open and extremely painful wound. The following points should be attended to: If possible, the flaps should be long and loose, so that they can be brought into apposition without tension; the sutures should not be drawn very tight, and if there is much bleeding, the needle may be passed through the mouth of the lateral vessels as recommended by Ashurst. Perchloride of mercury or carbolic lotion should be freely applied during the operation and the *whole* finger well cleaned, not merely the part near the wound. If the amputation is at the metacarpo-phalangeal joint, the hand should be well washed in antiseptic lotion. If only the terminal phalanx is cut off, the tendon of the flexors should be carefully stitched to the sheath or neighbouring structures; if this it not attended to the second phalanx becomes stiff upon the proximal one, and the stump is in the way. Hence the practice of some surgeons of always taking off the last two phalanges when the terminal one requires removal. In the fore-finger an attempt should be made to save as much as possible. It is a question whether the end of the proximal phalanx should be removed in amputations through the joints, but unless there is plenty of skin to make large, healthy flaps, it is certainly better to do so. After the dressings are applied, the hand should be firmly (not tightly) bandaged to ensure quiet, and a dose of chloral or nepenthe should be administered. If there is moderate freedom from pain, and no extension of tenderness up the arm, the parts should not be dressed for at least three or four days. The wound, it is needless to say, should be properly drained, and the arm kept in a sling.

There is often more distress, and even constitutional disturbance, after these small operations than after the amputation of a leg, and as much care should be taken as in the major operation to ensure good results.

